# Hepatitis A, B and C seropositivity among first-year healthcare students in western Turkey: a seroprevalence study

**DOI:** 10.1186/s12879-020-05247-5

**Published:** 2020-07-22

**Authors:** Ayla Acikgoz, Dilek Cimrin, Servet Kizildag, Nuran Esen, Pinar Balci, Ayca Arzu Sayiner

**Affiliations:** 1 Vocational School of Healthcare, Dokuz Eylul University, Balcova, 35340 Izmir, Turkey; 2 Department of Microbiology, School of Medicine, Dokuz Eylul University, Izmir, Turkey; 3 Department of Radiology, School of Medicine, Dokuz Eylul University, Izmir, Turkey

**Keywords:** Hepatitis A, Hepatitis B, Seroprevalence, Healthcare students

## Abstract

**Background:**

The risk of viral hepatitis among healthcare students (HCSs) is greater than that among the general population. Therefore, this study was conducted to investigate the seroprevalence of the hepatitis A virus (HAV), hepatitis B virus (HBV) and hepatitis C virus (HCV) among first-year HCSs at a university in Turkey and as a secondary objective, to determine the factors associated with HAV and HBV seropositivity.

**Methods:**

This cross-sectional study was performed in first-year HCSs in Izmir, western Turkey. Data were collected using a self-administered questionnaire including items on sociodemographic characteristics, medical history, and hygiene. A total of 650 HCSs were tested for the HAV, HBV and HCV markers. Categorical variables were compared using the chi-square test. The association between independent variables and anti-HAV seropositivity and anti-HBs seropositivity was assessed by multinomial logistic regression analysis.

**Results:**

The overall frequency of total anti-HAV seropositivity was 34.9%. HBsAg, total anti-HBc and anti-HBs seropositivity were found in 0.3, 1.2 and 93.7% of samples, respectively. All of the HCSs were negative for anti-HCV. Total anti-HAV seropositivity was found to be 1.73 times higher in those ≥21 years old, and it was 1.61 times higher in those who perceived their economic status to be average and 2.75 times higher in those who perceived their economic status to be low. Total anti-HAV seropositivity was found to be 4.37 times higher in those who lived in provinces with intermediate human development index levels. Total anti-HBs seropositivity was found to be 2.48 times higher in those ≤20 years old, and it was 2.13 times higher in those who perceived their economic status to be average.

**Conclusions:**

Approximately two out of three HCSs were susceptible to HAV infection. Since HCSs are at high risk for HAV infection, they should be vaccinated before medical clerkships begin. Our results indicate that there is a high prevalence of anti-HBs seropositivity among HCSs. This result may be largely attributed to the implementation of a successful vaccination program in Turkey since 1998.

## Background

Health care workers (HCWs) may be exposed to viruses such as the hepatitis B virus (HBV) and hepatitis C virus (HCV) in hospital environments. There are various contamination routes, such as accidental needlestick injury or exposure to infected blood, semen, and other body fluids [1, 2]. Since healthcare students (HCSs) spend periods of time in hospitals during their education, they are at risk for hepatitis virus infections.

Hepatitis A virus (HAV) causes acute viral hepatitis. The reported incidence of HAV infection is approximately 1.4 million cases worldwide. While hepatitis A is often asymptomatic in children, it can become a serious, even deadly, disease in adolescents and adults [3]**.**

Improvements in hygiene and socioeconomic conditions worldwide have resulted in lower disease incidence. HAV infection has moderate endemicity in Turkey [4, 5]. It is emphasized by the World Health Organization (WHO) that comprehensive vaccination studies are useful in countries with moderate endemic levels for HAV infection incidence [4]. HAV infection is a vaccine-preventable disease that can be widely prevented by immunization strategies during childhood. Since 2012, the Ministry of Health has added the HAV vaccine to the mandatory childhood vaccine schedule in Turkey. The HAV is primarily transmitted through water, food and direct contact with an infected person. Theoretically, HCWs have a higher chance of direct contact with patients infected with hepatitis A and are at a higher risk than the general population. The HAV vaccine is recommended for HCWs in Turkey by the Ministry of Health [6]. Students who have studied in the health field have the potential to come into contact with patients carrying contagious diseases. Therefore, HCSs who have not had HAV infection or vaccination are at risk for HAV infection [1]. Either at the beginning of education or before clinical training, HCSs should be screened for hepatitis A.

The HBV and HCV are among the main causes of liver cirrhosis and hepatocellular carcinoma [1, 3]. These viruses cause public health problems worldwide. The WHO estimated that there were 257 million people with chronic HBV infection and 71 million people with chronic HCV infection worldwide in 2015. The prevalence of HBV infection is 3.5% worldwide [3]. The prevalence of HBV in Turkey varies according to the region (2–8%) and increases from west to east. The prevalence of HBV in the general population in Turkey is higher than that in European countries [7–9].

In the past three decades, public health programs to control viral hepatitis infections have been carried out successfully [3]. One of the most effective public health programs to prevent HBV infection is the HBV vaccination program [1]**.** The national HBV vaccination program in Turkey began in July 1998. The Ministry of Health has added the HBV vaccine to the mandatory childhood vaccine schedule in Turkey.

HCSs, like HCWs, may be at risk for HBV and HCV infections due to the increased threat of needlestick injury. The HBV vaccine is recommended in high-risk groups, such as HCWs and HCSs [1]. In Turkey, it is obligatory for HCWs to undergo a hepatitis B serology screening before starting their practice and to get the hepatitis B vaccine if they are negative for anti-HBs. However, such a requirement is not yet available for HCSs.

Vaccination of HCSs is important to protect HCSs from hepatitis virus infections. However, it is unclear whether HCSs are vaccinated against hepatitis viruses. Therefore, this study was conducted to investigate the seroprevalence of HAV, HBV, and HCV among first-year HCSs in a university in Turkey and, as a secondary objective, to determine the factors associated with HAV and HBV seropositivity.

## Methods

### Study population

This cross-sectional study was performed between April 2018 and July 2018 in first-year HCSs at Dokuz Eylul University Health Campus in the following schools: School of Physical Therapy and Rehabilitation, Faculty of Nursing, and Vocational School of Healthcare. The Dokuz Eylul University Health Campus is in Izmir, Western Turkey. All first-year students were invited to participate in the study, with no sampling. Ninety-eight students declined to participate in the study. As a result, a total of 650 first-year HCSs participated in this study.

### Data collection

Data were collected using a self-administered questionnaire, which included sociodemographic characteristics, self-reported vaccination history against HAV and HBV, perceived economic position, place of residence before attending university, main source of water in the household, residence in a house that had a sewage system, and presence of an HCW in the household. The provinces where the students lived before attending university were used for calculating the Human Development Index (HDI).

### Sample collection and laboratory analysis

Venous blood samples (7–9 ml) were collected from every participant. Samples were centrifuged at 10,000 rpm for 10 min, and the sera were removed. During the measurements, laboratory personnel were blinded.

The serological markers were detected by enzyme-linked immunosorbent assay (ELISA) using Architect kits on an Architect plus i2000 SR (Architect Systems and Abbot Diagnostics Division, USA).

Total anti-HAV S/CO values ≥1.0, HBsAg S/CO values ≥1.0, anti-HBs concentrations ≥10 mIU/mL, total anti-HBc S/CO values ≥1.0, anti-HCV S/CO values ≥ 1.0 were considered positive, and samples below these thresholds were considered negative according to the manufacturer’s instructions.

### Ethical considerations

The study was conducted in accordance with the principles of the Helsinki Declaration. Ethical approval was obtained from the Clinical Research Ethics Committee of Dokuz Eylül University, Izmir, Turkey. Participants were informed of their rights, including the right to refuse to sign the consent form, the right to withdraw their consent from the study at any time, and the right to participant confidentiality, before signing the informed consent form.

### Statistical analysis

Continuous variables are presented as the mean and standard deviation. Categorical variables are presented as a number and frequency and 95% confidence intervals (95% CI) and compared using the chi-square test. The association between independent variables that were found to be statistically significant by the chi-square test and anti-HAV seropositivity and anti-HBs seropositivity was assessed by multinomial logistic regression analysis. Statistical analyses were performed using Statistical Package for Social Sciences Version 20.0 (IBM Corp., Armonk, NY, USA). A value of *p* < 0.05 was considered statistically significant.

## Results

A total of 650 HCSs participated in the study and were tested for HAV, HBV and HCV markers. The majority of students were female (*n* = 432, 66.5%). The age of the HCSs ranged from 18 to 37 years with an average of 19.8 ± 2.1 years. HCSs included 77 physiotherapy students (11.8%), 234 nursing students (36.0%) and 339 vocational school students (52.2%).

The seropositivity for total anti-HAV in HCSs was 34.9%. HBsAg was detected in two students (0.3%) in this study. Only 1.2% of the students were serologically positive for total anti-HBc. Six hundred and nine (93.7%) of the participants were found to be positive for anti-HBs. As a result, 6.3% of the students were serologically negative for anti-HBs, indicating that they were not immune and were susceptible to infection. All of the HCSs were negative for anti-HCV (Table 1).

**Table 1:** Prevalence of hepatitis A, B and C seropositivity according to sex among healthcare students (*n* = 650)

Sex	Total anti- HAV n (%)	HBsAg n (%)	Total anti-HBc n (%)	Anti-HBs n (%)	Anti-HCV n (%)
Male	100 (45.9)*	0	5 (2.3)	208 (95.4)	0
Female	127 (29.4)	2 (0.5)	3 (0.7)	401 (92.8)	0
Total n (%)	227 (34.9)	2 (0.3)	8 (1.2)	609 (93.7)	0

*p = 0.001

We observed significantly higher anti-HAV seropositive results in males than in females. In addition, the prevalence of anti-HAV seropositivity was significantly higher in students ≥21 years, in low and average perceived economic position groups, and in those living in provinces with intermediate HDI levels before attending university (*p* < 0.05). Total anti-HAV seropositivity was higher in subjects who lived in a house without a sewage system (*p* < 0.05) (Table 2).

**Table 2:** Association between demographic features and the prevalence of total anti-HAV seropositivity among healthcare students (*n* = 650)

Variables		Total n	Positive n (%)	Negative n (%)	Odds Ratio (CI 95%)	***p***-value
**Sex**	Male	218	100 (45.9)	118 (54.1)	2.03 (1.45–2.85)	**< 0.001**
	Female	432	127 (29.4)	305 (70.6)	Ref.	
**Age group (years)**	≥21	93	42 (45.2)	51 (54.8)	1.65 (1.05–2.58)	**0.014**
	≤20	557	185 (33.2)	372 (66.8)	Ref.	
**Perceived economic position**	Low	52	30 (57.7)	22 (42.3)	4.81 (2.45–9.58)	**< 0.001**
	Average	452	165 (36.5)	287 (63.5)	2.04 (1.33–3.20)	**< 0.001**
	High	146	32 (21.9)	114 (78.1)	Ref.	
**Water source**	City water	46	18 (39.1)	28 (60.9)	1.20 (0.56–2.51)	0.313
	Licenced-wrapped water	515	178 (34.6)	337 (65.4)	0.98 (0.61–1.59)	0.477
	Well water	89	31 (34.8)	58 (65.2)	Ref.	
**House that has the sewage system**	No	134	63 (47.0)	71 (53.0)	1.90 (1.29–2.80)	**< 0.001**
	Yes	516	164 (31.8)	352 (68.2)	Ref.	
**Childhood residence**	Districts	275	93 (33.8)	182 (66.2)	1.02 (0.70–1.51)	0.441
	Country	170	66 (38.8)	104 (61.2)	1.27 (0.83–1.95)	0.129
	Province	205	68 (33.2)	137 (66.8)	Ref.	
**Human development index**	Intermediate	218	132 (60.6)	86 (39.4)	5.42 (3.81–7.76)	**< 0.001**
	High	432	95 (22.0)	337 (78.0)	Ref.	
**Self-reported HAV vaccination history**	No	102	44 (43.1)	58 (56.9)	1.38 (0.72–2.65)	0.162
	Unknown	483	160 (33.1)	323 (66.9)	0.90 (0.52–1.57)	0.356
	Yes	65	23 (35.4)	42 (64.6)	Ref.	

The prevalence of anti-HBs seropositivity was significantly higher in students ≤20 years, in low and average perceived economic position groups, in those who lived in provinces with intermediate HDI levels before attending university and in those with a self-reported HBV vaccination history (*p* < 0.05). There was no association between anti-HBs seropositivity and sex or area of childhood residence (Table 3).

**Table 3:** Association between subject characteristics and the prevalence of anti-HBs seropositivity among healthcare students (*n* = 650)

Variable		Total n	Positive n (%)	Negative n (%)	Odds Ratio (CI 95%)	***p***-value
**Sex**	Female	432	401 (92.8)	31 (7.2)	Ref.	
	Male	218	208 (95.4)	10 (4.6)	1.60 (0.78–3.49)	0.202
**Age group (years)**	≥21	81	81 (87.1)	12 (12.9)	Ref.	
	≤20	557	528 (94.8)	29 (5.2)	2.69 (1.27–5.43	**0.010**
**Perceived economic position**	High	146	130 (89.0)	16 (11.0)	Ref.	
	Average	452	428 (94.7)	24 (5.3)	2.19 (1.10–4.24)	**0.024**
	Low	52	51 (98.1)	1 (1.9)	6.24 (1.07–135.4)	**0.038**
**Childhood residence**	Province	205	188 (91.7)	17 (8.3)	Ref.	
	Districts	275	263 (95.6)	12 (4.4)	1.97 (0.92–4.35)	0.080
	Country	170	158 (92.9)	12 (7.1)	1.19 (0.55–2.63)	0.665
**Presence of an HCW in the household**	No	549	513 (93.4)	36 (6.6)	Ref.	
	Yes	101	96 (95.0)	5 (5.0)	1.34 (0.54–3.95)	0.570
**Human development index**	High	432	398 (92.1)	34 (7.9)	Ref.	
	Intermediate	218	211 (96.8)	7 (3.2)	2.57 (1.16–6.36)	**0.017**
**Self-reported HBV vaccination history**	No	77	69 (89.6)	8 (10.4)	Ref.	
	Yes	194	191 (98.5)	3 (1.5)	7.31 (1.94–34.94)	**0.002**
	Unknown	379	349 (92.1)	30 (7.9)	1.34 (0.55–2.99)	0.472

Total anti-HAV seropositivity was found to be 1.73 times higher in those ≥21 years old than in those ≤20 years old (OR: 1.73, 95% CI = 1.05–1.86), and it was 1.61 times higher in those who perceived their economic status to be average (OR: 1.61, 95% CI = 1.01–2.58) and 2.75 times higher in those who perceived their economic status to be low (OR: 2.75, 95% CI = 1.31–5.79) than in those who perceived their economic status to be high. Total anti-HAV seropositivity was found to be 4.37 times higher in those who lived in provinces with intermediate HDI levels before attending university than in those living in provinces with high HDI levels before attending university (OR: 4.37, 95% CI = 3.00–6.38) (Table 4).

**Table 4:** Association between subject characteristics and total anti-HAV seropositivity and anti-HBs seropositivity among healthcare students (*n* = 650)

Variables		Total Anti-HAV seropositivity Adj. OR ^a^ (CI 95%)	p	Anti-HBs seropositivity Adj. OR^b^ (CI 95%)	p
**Sex**	Female	Ref		**–**	
	Male	1.44 (0.98–2.10)	0.057	–	
**Age group (years)**	≤20	Ref		2.48 (1.16–5.29)	**0.018**
	≥21	1.73 (1.05–1.86)	**0.030**	Ref	
**Perceived economic position**	High	Ref		Ref	
	Average	1.61 (1.01–2.58)	**0.046**	2.13 (1.07–4.24)	**0.030**
	Low	2.75 (1.31–5.79)	**0.007**	5.41 (0.68–43.04)	0.110
**House that has the sewage system**	Yes	Ref		–	
	No	1.38 (0.90–2.13)	0.135	–	
**Human development index**	High	Ref		Ref	
	Intermediate	4.37 (3.00–6.38)	**< 0.001**	2.32 (0.97–5.51)	0.057
**Self-reported HBV vaccination history**	No	–		Ref	
	Yes	**–**		8.23 (2.08–32.62)	**0.003**
	Unknown	**–**		1.43 (0.61–3.35)	0.400

^a^Adjusted according to sex, age, perceived economic position, a house that has the sewage system and human development index

^b^Adjusted according to age, perceived economic position, human development index and self-reported HBV vaccination history

Total anti-HBs seropositivity was found to be 2.48 times higher in those ≤20 years old than in those ≥21 years old (OR: 2.48, 95% CI = 1.16–5.29), and it was 2.13 times higher in those who perceived their economic status to be average than in those who perceived their economic status to be high (OR: 2.13, 95% CI = 1.07–4.24). Total anti-HBs seropositivity was found to be 8.23 times higher in those who reported HBV vaccination history than in those who did not report a vaccination history (OR: 8.23, 95% CI = 2.08–32.62) (Table 4).

## Discussion

A total of 650 HCSs participated in this study and were tested for the HAV, HBV and HCV markers. The seropositivity for total anti-HAV in HCSs was 34.9%. Most of the HCSs (93.7%) were anti-HBs positive. All of the HCSs were anti-HCV negative. The ratio of anti-HAV seropositivity was higher in those ≥21 years old, in those who perceived their economic status to be average and low, and in those who lived in provinces with intermediate HDI levels before attending university. The ratio of total anti-HBs positivity was higher in those ≤20 years old, in those who perceived their economic status to be average and in those who reported a vaccination history against HBV.

In our study, approximately two out of three students were not immune to the HAV. In similar studies in Turkey, anti-HAV seropositivity was reported as 51% [10], 27.3% [11] and 15% [12]. In Korea, anti-HAV seropositivity was reported as 11.4% among 1st- to 3rd-year medical school students [13], and in medical students in Iran, the prevalence of anti-HAV seropositivity was 70% in one study [14] and 34% in another study [15]. In another study from Italy, 3.16% of nursing students were immune to the HAV [16]. These differences between studies may be attributed to the health behaviors of participants, hygiene, sanitary conditions, and socioeconomic levels of the country.

The incidence rates of HAV infection decrease in children and adolescents, and the risk of HAV infection increases in adults. Serious and severe complications of HAV infection are more common in adults [4]. It is known that the anti-HAV seropositivity rate is associated with increasing age [17]. In our study, the prevalence of anti-HAV seropositivity was significantly higher in students 21 years old and older. However, our results showed that a large number of HCSs lacked natural immunity to HAV infection and were susceptible to the virus. Anti-HAV seropositivity in this study was relatively low compared with that in a study conducted in medical students in the same region in 2005, in which the seroprevalence rate was 64% [18]. We predict that the average age for HAV infection will shift to older ages with the incrementally changing standard of living in our country. Therefore, the number of susceptible students in the coming years might increase. Since HCSs are at high risk for HAV infection, they should be vaccinated before medical clerkship begins.

In this study, using a univariate analysis (chi-square test), it was determined that anti-HAV seropositivity was significantly higher among male students than among female students. This result was not in agreement with the observations reported in other studies [12, 14, 16, 19]. We have no explanation for this finding. Moreover, this relationship was not observed in the logistic regression analysis.

We found higher total anti-HAV seropositivity in subjects who had lived in a house that did not have a sewage system. Water contaminated with feces from infected humans can potentially spread the HAV [4]. Waterborne transmission is usually associated with sewage-contaminated water. This finding was not statistically significant in the logistic regression analysis.

We found a relationship between anti-HAV seropositivity and socioeconomic indicators, including the HDI and perceived economic position. In our study, low socioeconomic status and an intermediate HDI were found to be the most important factors related to anti-HAV positivity. In a recent systematic review published in 2017, it was found that economic variables appear to be much better predictors of HAV prevalence than water and sanitation indicators. There is a strong negative correlation between the HDI and HAV prevalence [17]. Our results, specifically the relationship between socioeconomic indicators and anti-HAV seropositivity, are consistent with the results of other studies conducted in medical students [15, 20, 21] and nursing students [12].

In our study, most students (93.7%) had immunity against HBV infection. Anti-HBs seropositivity was found in 31.9% in a study conducted in the general Turkish population in 2009–2010 [8]. The higher anti-HBs seropositivity in our results may be due to the young age of the students. To control HBV infection, many countries have added hepatitis B vaccination to the expanded program on immunization (EPI). In Turkey, hepatitis B vaccination was added to the EPI in 1998. Moreover, in our study, the anti-HBs seropositivity was 2.7 times higher in students ≤20 years of age than in those older than 20 years of age. These findings can be attributed to the effectiveness of the vaccination program. In our study, the percentage of anti-HBs seropositive HCS was consistent with those reported in some other countries, such as Poland, Mexico, the United States, and Italy [22–25]; nevertheless, it was higher than the results of studies from Saudi Arabia, Taiwan, the United Arab Emirates, Italy, Spain, Palestine, and Iran [21, 26–31]. The anti-HB seropositivity of HCSs is widely distributed (17.0 to 89.4%) in Turkey [12, 32, 33]. Variation in the prevalence of anti-HBs seropositivity in previous studies conducted in different countries, and even within the same country, may be attributed to differences in the study groups (e.g., first-year students or all students), data collection dates (some studies were conducted in participants who born before the introduction of the hepatitis B vaccine to the EPI), compliance with the immunization program, and socioeconomic status.

In our study, the prevalence of anti-HBs seropositivity was higher in low and intermediate economic groups. We have no explanation for this finding. This relationship was not observed in the low economic group in the logistic regression analysis. Moreover, the 95% confidence intervals for the results were very large. Therefore, the results should be considered with these limitations in mind. In a population-based study conducted in Turkey, young participants (aged 2–21 years old) with low income were more likely to be vaccinated; inversely, old participants (22–89 years old) with high income were more likely to be vaccinated [9]. Our results and these results may reflect the strength of primary healthcare and the effectiveness of the immunization program in the past 20 years. Since 1998, HBV vaccination for newborns has been done free of charge in Turkey. Therefore, those with low income may be better able to comply with the vaccination program. Furthermore, HBV vaccination has been performed for nonvaccinated children at primary and high schools.

In our study, the prevalence of anti-HBs positivity was significantly higher in participants who declared being vaccinated against HBV. However, most HCSs (53.2%) did not remember whether they were vaccinated. Therefore, it may be more accurate to examine anti-HB seropositivity to decide on whether to vaccinate. The 41 unvaccinated HBsAg/anti-HBs/anti-HBc seronegative HCSs were immunized with three doses of the hepatitis B vaccine.

Anti-HCV seropositivity was not detected in the HCSs in our study. The prevalence of HCV infection in Turkey is low (approximately 1%). This might be related to rigorous screening of blood donors for HBV and HCV and the low rate of intravenous drug use in Turkey [8]. Our result was consistent with other studies conducted in Saudi Arabia, the United Arab Emirates, Brazil, Palestine, and Turkey [12, 21, 26, 33, 34]. According to these results, HCV infection was not an important health issue among HCSs.

The study limitations are mainly related to data collection. We collected self-reported data regarding sociodemographic characteristics, vaccination history, perceived economic position, place of residence, source of water, and sewage system status. Although self-reporting is an acceptable method of data collection in public health studies, it is associated with potential information bias.

## Conclusion

Approximately two out of three HCSs were susceptible to HAV infection. Since HAV infection can become a serious and even deadly disease in adolescents and adults and HCSs are at high risk for HAV infection, they should be vaccinated before medical clerkships begin. This study showed a high prevalence of anti-HBs seropositivity among HCSs. This result may be largely attributed to the implementation of a successful vaccination program in Turkey during recent decades. Mandatory vaccination should be considered for HCSs before they start their clinical practice.

## Data Availability

The datasets used during the current study are available from the corresponding authors upon reasonable request and with permission from the University of Clinical Research Ethics Committee.

## Abbreviations

HAV: Hepatitis A virus
HBV: Hepatitis B virus
HCV: Hepatitis C virus
HCSs: Healthcare students
HCWs: Health care workers
HDI: Human development index
WHO: World Health Organization
